# Supplementary material for: The Inferential Binding Sites of GCGR for Small Molecules Using Protein Dynamic Conformations and Crystal Structures
Source: Int J Mol Sci. 2024 Aug 1;25(15):8389. doi: 10.3390/ijms25158389 (PMC11313378; doi:10.3390/ijms25158389)
Supplement: Supplementary file 1 [file ijms-25-08389-s001.zip › ijms-3057215-supplementary.pdf]

Table S1

Table S1. The specific situation of each small molecule MD simulation

Molecule	Pocket	System (abbreviation)	Time（ns）
MK-0893	Pocket 2	MK8-1、MK8-2	100
	Pocket 2	MK8-3	90
Bay 27-9955	Pocket 3	Bay	180
MK-3577	Pocket 2	MK	100
	Pocket 4	MK-p	180
LY2409021	Pocket 2	LY-1、LY-2	100
	Pocket 2	LY-3	90
	Pocket 5	LY-p	100
PF-06291874	Pocket 2	PF-1	90
	Pocket 2	PF-2、PF-3	100
LGD-6972	Pocket 5	LGD	130

Figure S1

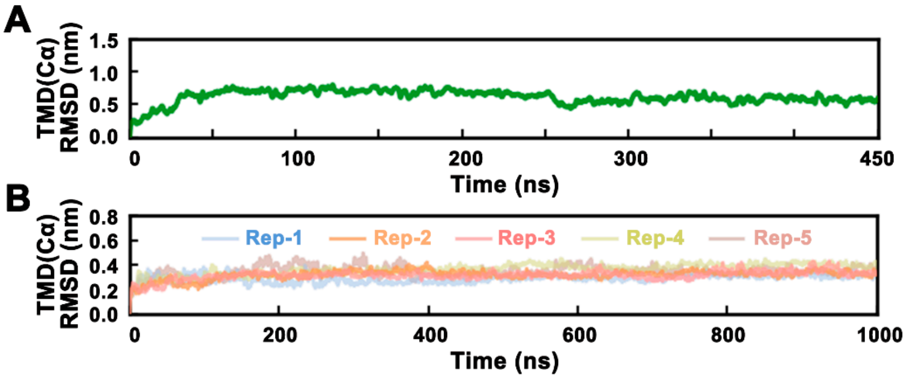

Figure S1. The RMSD of GCGR TMD Cα over time in GCGR system (A) and GCGR/glucagon system (B).

Figure S2

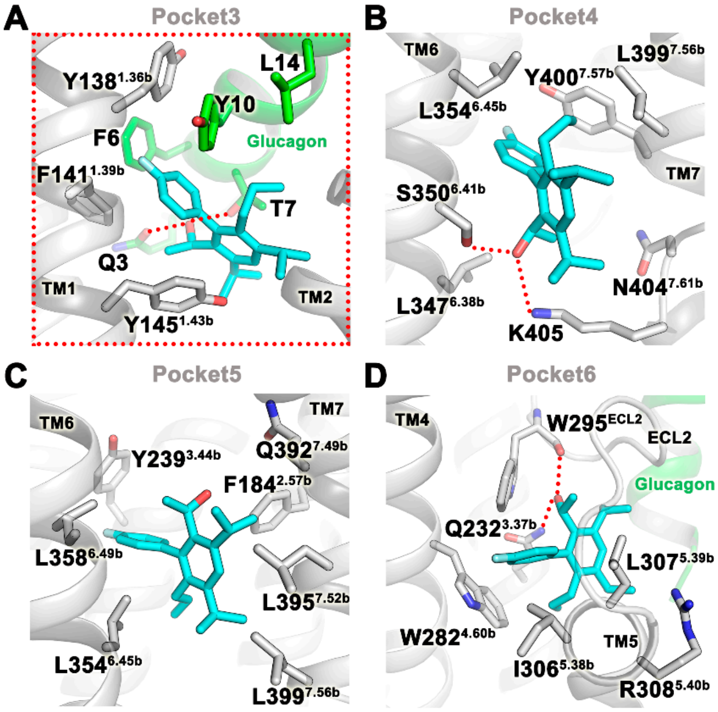

Figure S2. Binding modes of Bay 27-9955 (cyan) in different pockets of GCGR (gray). Glucagon is shown in green cartoon, and hydrogen bonds are shown in red dashed lines. The docking conformation selected for MD simulation is marked with red box.

Figure S3

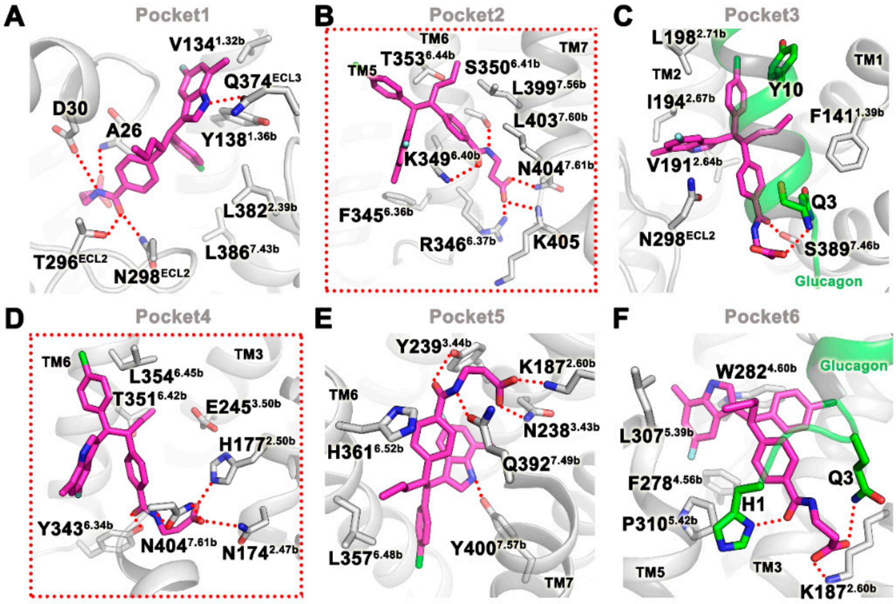

Figure S3. Binding modes of MK-3577 (magenta) in different pockets of GCGR (gray). Glucagon is shown in green cartoon, and hydrogen bonds are shown in red dashed lines. The docking conformations selected for MD simulations are marked with red boxes.

Figure S4

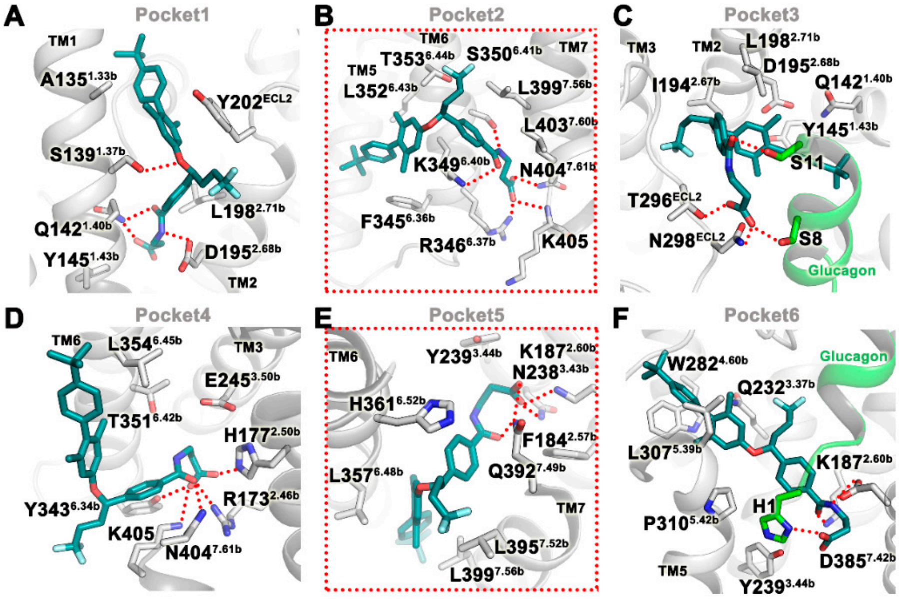

Figure S4. Binding modes of LY2409021 (deepteal) in different pockets of GCGR (gray). Glucagon is shown in green cartoon, and hydrogen bonds are shown in red dashed lines. The docking conformations selected for MD simulations are marked with red boxes.

Figure S5

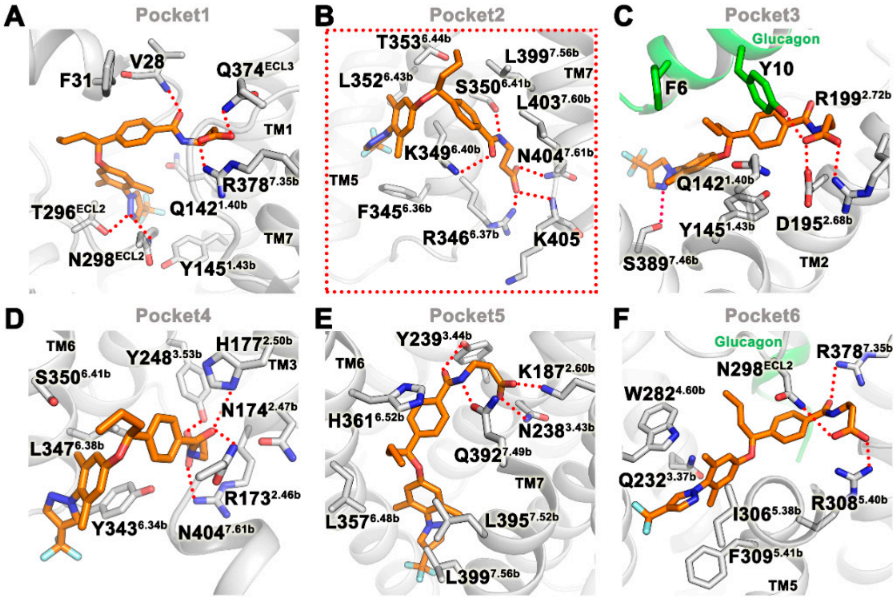

Figure S5. Binding modes of PF-06291874 (orange) in different pockets of GCGR (gray). Glucagon is shown in green cartoon, and hydrogen bonds are shown in red dashed lines. The docking conformation selected for MD simulation is marked with red box.

Figure S6

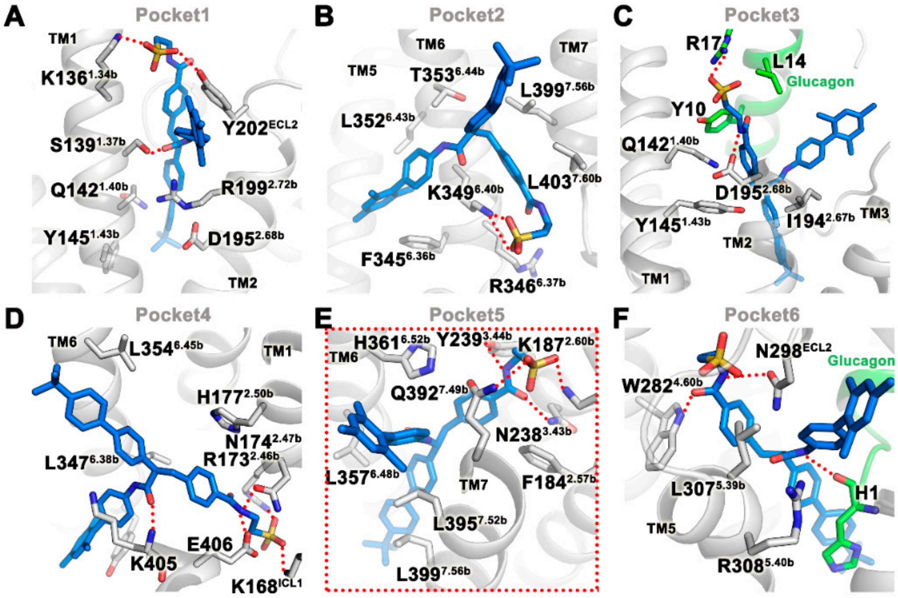

Figure S6. Binding modes of LGD-6972 (marine) in different pockets of GCGR (gray). Glucagon is shown in green cartoon, and hydrogen bonds are shown in red dashed lines. The docking conformation selected for MD simulation is marked with red box.

Figure S7

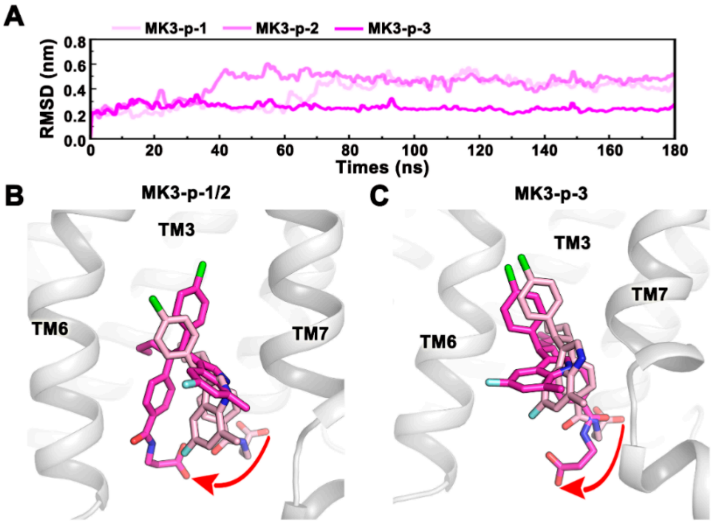

Figure S7. MD simulation indicated instability of MK-3577 binding in Pocket 4. (A) RMSD of MK-3577 over time in each simulated trajectory. (B-C) Comparison between representative conformations (magenta) of MD simulation and initial conformation (pink). Red arrows indicated the tendency of conformational changes.
